# Impact of COVID-19 Era on the Anterior Cruciate Ligament Injury Rehabilitation: A Scoping Review

**DOI:** 10.3390/jcm12175655

**Published:** 2023-08-30

**Authors:** Nicola Marotta, Alessandro de Sire, Dario Calafiore, Francesco Agostini, Lorenzo Lippi, Claudio Curci, Francesco Ferraro, Andrea Bernetti, Marco Invernizzi, Antonio Ammendolia

**Affiliations:** 1 Department of Experimental and Clinical Medicine, University of Catanzaro “Magna Graecia”, 88100 Catanzaro, Italy; nicola.marotta@unicz.it; 2 Research Center on Musculoskeletal Health, MusculoSkeletalHealth@UMG, University of Catanzaro “Magna Graecia”, 88100 Catanzaro, Italy; ammendolia@unicz.it; 3 Department of Medical and Surgical Sciences, University of Catanzaro “Magna Graecia”, 88100 Catanzaro, Italy; 4 Physical Medicine and Rehabilitation Unit, Department of Neurosciences, ASST Carlo Poma, 46100 Mantova, Italy; dario.calafiore@asst-mantova.it (D.C.); francesco.ferraro@asst-mantova.it (F.F.); 5 Department of Anatomical and Histological Sciences, Legal Medicine and Orthopedics, Sapienza University, 00185 Rome, Italy; 6 Department of Neurological and Rehabilitation Science, IRCCS San Raffaele, 00163 Rome, Italy; 7 Physical and Rehabilitative Medicine, Department of Health Sciences, University of Eastern Piedmont “A. Avogadro”, 28100 Novara, Italy; lorenzolippi.mt@gmail.com (L.L.); marco.invernizzi@med.uniupo.it (M.I.); 8 Translational Medicine, Dipartimento Attività Integrate Ricerca e Innovazione (DAIRI), Azienda Ospedaliera SS, Antonio e Biagio e Cesare Arrigo, 15121 Alessandria, Italy; 9 Department of Biological and Environmental Sciences and Technologies (DiSTeBA), University of Salento, 73100 Lecce, Italy; andreabernetti@gmail.com

**Keywords:** COVID-19, physiotherapy, sports, rehabilitation, anterior cruciate ligament injury, knee, ACL

## Abstract

The surgical intervention rate and the subsequent rehabilitation plan for anterior cruciate ligament (ACL) injury was crucially affected by the COVID-19 pandemic due to the necessity in the face of the emergency. This review aimed to evaluate potential persistent and residual symptoms after COVID-19 disease, including fatigue and neuromuscular disorders. A scoping review design and methodology were used due to the exploratory nature of the research question, according to literature searches on PubMed/Medline, Scopus, Web of Science (WoS), and Physiotherapy Evidence Database (PEDro) electronic databases using the following keywords: “Anterior Cruciate Ligament”, “ACL”, “SARS-CoV-2”, and “COVID-19”. Undertraining and muscular knee imbalance might cause inefficient movement strategies, lack of knee stability, and increasing load with negative implications in ACL injuries. In the post-surgery period, during COVID-19, telerehabilitation approaches appeared to be successfully applied to maintain strength and range of motion in this condition. However, no definitive data are available regarding the most effective interventions. This scoping review showed the influence of the COVID-19 pandemic and associated restrictions on postoperative and rehabilitative care of ACL injuries.

## 1. Introduction

The COVID-19 pandemic has recently spread worldwide with a negative impact on all forms of sport, especially in its first wave [1]. Since the onset of the pandemic in March 2020, the number of sports injuries has decreased significantly, and the proportion of sport-related injuries fell during COVID-19 largely due to modified or canceled sports seasons [2]. In this scenario, COVID-19 caused rapid changes in the standard of care and implementation of telemedicine for many sports medicine providers. However, many other challenges persisted for these patients, including limited access to in-person rehabilitation services, restricted access to weight rooms and training facilities, and a lack of resources to perform sport-specific activities [3].

Anterior cruciate ligament (ACL) injury is one of the most frequent and significant sport-related diseases, with a high prevalence in young and active individuals [4]. A Google trends analysis showed that from March 2020 to May 2020, the terms “ACL reconstruction”, “ACL repair”, and “anterior cruciate ligament reconstruction” were searched significantly less often than in the pre-pandemic period, showing a reduced interest in the general population [3]. ACL rupture is one of the more common sports injuries, and 75% of high school athletes are likely to undergo surgical intervention with the aim of restoring knee stability [5]. Though patients who undergo ACL reconstruction (ACLR) showed positive outcomes in terms of knee function and symptoms compared to non-surgically treated patients, outcomes have been far from satisfying during the COVID-19 pandemic [6]. Moreover, ACL injury is related to a higher risk of knee re-injury and long-term disabilities (i.e., early osteoarthritis) that should be effectively targeted using a specific rehabilitation approach [7,8].

In this scenario, it has not been clarified what effects the COVID-19 pandemic and related contact restrictions have had on postoperative care, nor are the consequences on the clinical outcome known, whether measures taken during the pandemic impacted physiotherapy and medical follow-up after ACL reconstructions and whether this negatively affected clinical outcomes [9,10,11]. In fact, pre- and post-surgical rehabilitation is of paramount importance to maximize postoperative outcomes for the full long-term restoration of function and resilience. Studies have shown the relevance of physiotherapy consistent with early initiation of therapy, treatment adherence, and regular progress monitoring [9]. Despite these considerations, it is unclear what effects the COVID-19 pandemic and associated contact restrictions have had on postoperative care, and the consequences for clinical outcomes are also unknown [12]. Nevertheless, non-urgent elective surgeries were also canceled to preserve personal protective equipment, minimize the use of hospital resources, and prioritize staff and beds in the expectation of a surge in COVID-19 hospital admissions. As a result, ACL reconstructions were canceled or delayed, and some form of restriction on non-urgent elective surgeries remained in place until late November 2020 [13].

The hypotheses were that the COVID-19 pandemic scenario with mandatory lockdown measures and an unexpected shift to self-guided and home-based rehabilitation could lengthen the timing for appropriate intervention, might increase the complication rate, and could not guarantee acceptable outcomes with a telerehabilitation approach.

Thus, the aim of this scoping review was to characterize the impact of the COVID-19 pandemic on ACL injury management, focusing on epidemiology, rehabilitative impact, appropriateness of conventional treatments, and consequences in terms of functional outcomes in patients with ACL injuries.

## 2. Materials and Methods

### 2.1. Literature Strategy

A scoping review design and methodology was used due to the exploratory nature of the research question, according to the Preferred Reporting Items for Systematic Reviews and Meta-Analyses Extension for Scoping Reviews (PRISMA-ScR) [14]. We conducted literature searches of the PubMed/Medline, Scopus, Web of Science (WoS), and Physiotherapy Evidence Database (PEDro) electronic databases using the following keywords: “Anterior Cruciate Ligament”, “ACL”, “SARS-CoV-2”, “COVID-19”. The search was conducted by humans and English-language peer-reviewed publications.

### 2.2. Study Identification

Two independent reviewers performed a literature search between September 2022 and December 2022 and screened the studies for eligibility, reviewing all titles and abstracts identified from the search strategy. In agreement with the predefined eligibility criteria, full-text studies for all potentially eligible records were obtained; accordingly, with the previously decided eligibility criteria, the reviewers independently revised the bibliography. If a consensus was not reached by collegial discussion, a third reviewer was asked.

### 2.3. Data Extraction

Data extraction for each eligible manuscript was achieved independently by two reviewers using a predetermined spreadsheet. Only studies assessing ACL injury in adult patients (aged > 18 years) in a pandemic context were considered. Exclusion criteria were: (i) languages other than English; (ii) studies without full text available; (iii) conference abstracts, masters, or doctorate theses. A narrative method was used for both data extraction and data synthesis. Both data extraction and synthesis were performed by two independent reviewers. If consensus was not reached by collegial discussion, a third reviewer was asked. The reference lists of relevant systematic reviews identified during the title and abstract screen were also hand-searched to identify any potentially eligible articles that may have been missed in the electronic database search.

### 2.4. Data Screening

The search resulted in a total of 333 eligible articles. After the removal of duplicates, 262 articles were screened. Full-text screening resulted in 201 articles that were assessed for eligibility. In total, we identified 33 records: 31 observational studies, 2 reviews, and no clinical interventional studies were identified.

## 3. Effect of COVID-19 on Anterior Cruciate Ligament Injury Rates

In March 2020, there was a decline in the prevalence of ACLRs due to halted participation in sports or potential delayed ACLR surgery for hospital safety measures [3]. Nevertheless, after April 2020, a subsequent increase in ACLR surgery was reported, probably linked to the previous reduction trend, similar to the tendency reported by other surgical interventions [15]. In parallel, Kiani et al. highlighted an increasing trend in pediatric patients who underwent ACL reconstruction from 2016 to 2021, but there were fewer during the COVID-19 pandemic [3].

These findings are in contrast with data about other relevant sports injuries. More in detail, a study by Sclafani et al. reported a pointedly higher hamstring, groin, calf, quadriceps, thigh, and total injury counts in the 2020–2021 regular National Football League (NFL) season compared to the previous season, perhaps due to the absence of the 2020–2021 NFL preseason [16]. Moreover, the rates of ACL injuries almost doubled in the 2020–2021 season compared to the 2018–2019 season, while the number of Achilles injuries was the same between seasons [17]. These data might be related to the progressive deconditioning linked to sports restrictions during the COVID-19 pandemic; on the other hand, the return to sport placed growing emphasis on reconditioning training with high-intensity programs, increasing the risk of musculoskeletal injuries [3]. However, there are also conflicting data on the initial impact of the restrictions. Allahabadi et al. reported an increased percentage of in-season ACL tears in the 2020 NFL season compared to 2014–2019; this is attributable to a frameshift in the consistent trend of injuries in the first month to return of competitive play, with 2020 being in the regular season in September as opposed to the preseason in August [17]. Nevertheless, injuries remain significantly higher in the preseason training practice than in the regular season, and snaps and games played at the time of ACL injury did not differ by season [17]. Despite this, Omari et al. showed that in the 2020 NFL season, the number of Achilles tendon and hamstring tendon injuries rose while the number of ACL injuries remained constant compared with the 2017 to 2019 seasons. In fact, injuries occurring during the first four games of the 2020 NFL season were consistent, with higher rates of injuries seen in the preseason in previous years [18].

Taken together, these results suggested that the heterogeneity of these reported data might be partly related to sports characteristics that affect the biomechanical load on musculoskeletal systems and neuromuscular response and potentially associated with the multilevel interactions between sports and athlete individual characteristics. Nevertheless, these data might point towards an increased risk of ACL injury, but this evidence remains controversial.

## 4. Organic and Functional Impact of COVID-19 on Anterior Cruciate Ligament Injury

A low access rate to rehabilitation was reported in patients undergoing ACLR in the 4 months prior to the COVID-19 pandemic onset in 2020, generally associated with lower physical performance and a significant worsening in mental health scores [19]. Participation in high-frequency self-directed therapy led to an improvement in Return to Sport after Injury (ACL-RSI) scale 1 year after surgery [13]. These findings have led to the hypothesis that online and virtual therapeutic solutions adopted during the pandemic might have positive implications for postoperative rehabilitation outcomes, especially for those patients with limited access to in-person therapy due to time, distance, financial resources, or insurance constraints [20,21].

On the other hand, it has been reported that COVID-19 disease might lead to neurological impairment by damaging the central and peripheric nervous system through dysregulated inflammatory stimuli, hypoxia, and direct lesion mechanisms [22]. More in detail, COVID-19 neuromuscular sequelae might be directly or indirectly connected to SARS-CoV-2 infection; hence, a COVID-19 affinity for neural tissue has been proposed, with the direct infection and injury of motor neurons and peripheral nerves reported two to three weeks after infection [23,24]. In accordance, a third of patients with other coronavirus infections complained of myalgia, high creatine kinase (CK) levels, and rhabdomyolysis, suggesting that the infection may induce viral myositis [23,24]. Moreover, high levels of LDH were found in subjects with myalgia and fatigue, and elevated levels of myoglobin were documented in severe COVID-19 [25]. Whether the increment of CK levels and myopathic damage might be induced by toxic developments of cytokines patterns, the viral infection of the muscle itself, or other unknown mechanisms is still unclear [26]. Nonetheless, data from muscle biopsies suggested crucial pathophysiologic accountability of severe immune activation typically featured in COVID-19 patients [23,24]. 

In this context, Weaver et al. assessed a sample of 60 adolescents undergoing ACLR surgery during the COVID-19 pandemic versus the pre-pandemic timeframe, reporting that the normalized quadriceps peak torque for the unaffected limb was significantly higher for those who underwent surgery during the COVID pandemic with a large effect size compared to those who underwent surgery pre-COVID [6,27]. There were no other group differences for normalized peak torque on the involved limb (quadriceps or hamstrings) or limb symmetry indices; likewise, no differences were observed between groups for the Pediatric International Knee Documentation Committee (Pedi-IKDC) and ACL-RSI scores [6].

Moreover, it has been reported that the electrophysiological modifications characterizing COVID-19 athletes could increase the ACL injury risk [4]. In this scenario, Nepal et al. underlined the crucial role of electromyography in the diagnosis of COVID-19-related myopathic and neuropathic damage [28,29]; moreover, Agergaard et al., employing quantitative EMG, reported myopathic modifications in 11 (55%) of 20 long-COVID-19 patients [30]. The authors hypothesized that myopathy rather than neuropathy might be a conceivable explanation for physical fatigue in long-term COVID-19 disorder, even in a non-hospitalized setting [30]. Moreover, it was reported that a significant delay in the muscular activation time of the Vastus Medialis (VM), Rectus Femoris (RF), and Medial and Lateral Hamstrings at the return to play, compared to muscular activation before COVID-19 [4]. The VM and RF, via the indirect relationship with the patellar ligament, provide a stabilizing torque in both varus and valgus knee necessities [31,32,33]. Consequently, a delay in quadricep muscle activation depicts a dynamic instability of the knee during the initial phase of the cutting motions, from the pre-contact to the acceptance of the weight phase [33,34,35]. Furthermore, Demir et al. showed that this period of confinement led to a decrease in hamstring eccentric strength, a decrease in posterior chain flexibility in the legs, and an increase in hamstring injury incidence shortly after these players returned to professional soccer training [36,37]. On the other hand, Sclafani et al. reported that musculoskeletal injuries are more likely the consequence of rare or unusual mechanisms instead of being associated with a decreased preseason training period with an abrupt rise in the “acute workload” of players who entered a rigorous NFL season, as these players had not appropriately increase their athletic workload gradually during a normal preseason [16].

Altogether, this evidence suggested that the COVID-19 pandemic might have direct and indirect effects on the skeletal muscle system and neuromuscular control. On the other hand, as for epidemiological data, even organically and functionally, the authors do not agree in identifying a clear cause-and-effect relationship in this scenario; therefore, a comprehensive assessment of COVID-19 sequelae is mandatory for tailoring a patient-centered prevention and rehabilitation approach to ACL injuries.

## 5. Role of Medical Prevention for Anterior Cruciate Ligament Injury

ACL ruptures are considered debilitating knee injuries and represent the injuries that receive the most attention in the sports medicine field [36]. However, ACL ruptures occur for over 50% of all knee injuries and most commonly require surgical treatment, resulting in high healthcare costs and a great impact on physical and psychological health [38,39]. Moreover, long-term consequences of ACL rupture include an up to 80% possibility of developing arthritis in the affected knee and, a second ACL injury to the reconstructed knee and injury to the contralateral knee, especially in athletes that return to participation in sport [40,41].

Neuromuscular control in ACL injury risk combined with an inherent ability to modify associated risk factors might play a pivotal role in ACL injury prevention [42]. In particular, multimodal prevention programs aim to reduce dynamic valgus and landing forces and optimize muscle activation and strength imbalances to decrease overall injury risk during jumping, landing, and cutting movements [43]. In 2020, a meta-analysis by Huang et al. demonstrated that the use of ACL injury prevention programs (IPPs), including plyometric, agility, and strengthening exercises, led to a 53% overall reduction in ACL injury rates [44]. In this scenario, plyometric activities have been shown to be one of the most effective components of prevention programs [43,44,45]. The preventative outcomes that result from plyometric training, especially landing stabilization exercises during a jump, are related to improved landing mechanics, and some studies have specifically observed a decrease in knee valgus and an increase in knee flexion at landing after implementing training [46,47].

On the other hand, the direct efficacy of agility exercises (AE) on ACL injury prevention is still debated, but several effective prevention programs include it as a training component. Brunner et al. affirmed that AE seems more impactful when the agility exercises are sport-specific and are performed in combination with plyometric, strength, and balance exercises as part of a multicomponent program [48]. The hamstring plays a significant role as an ACL agonist in preventing anterior tibial translation achieved by a higher ratio of hamstrings to quadriceps activity, which can be increased by eccentric strengthening exercises [49]. Regarding the gluteus maximus and medius, optimizing muscle strength is important for decreasing femoral rotation and dynamic knee valgus during high-risk movements [50]. Moreover, proprioceptive deficits can induce unwanted strain on the ACL caused by interruption of proper muscle activation. To improve these deficits, efficient balance exercises should be aimed to increase dynamic stability and improve knee posture and control while in unstable positions, enhancing the outcome of an injury prevention program when combined with other types of exercises [51].

Despite the efficacy of complex multimodal ACL injury prevention programs, ACL injury rates have not successfully improved, primarily due to a lack of implementing programs. An increasing public awareness and knowledge of the benefits of utilizing these programs can bridge the gap between research on the topic and standard use of prevention strategies and successfully reduce ACL injuries in athletes [52,53]. In addition to encouraging the implementation of individual prevention programs, applying screening tests as a clinical standard early on and throughout athletes’ careers has the potential to be a crucial component of targeted injury prevention [54,55]. Furthermore, fatigue and overtraining without proper rest and recovery have been shown to increase the risk of ACL injury [56]. Recently, Snyder et al. observed significant alterations in physical performance and biomechanical risk factors related to ACL injury in athletes participating in two soccer matches with less than 48 h of rest in between [57].

**Figure 1:**
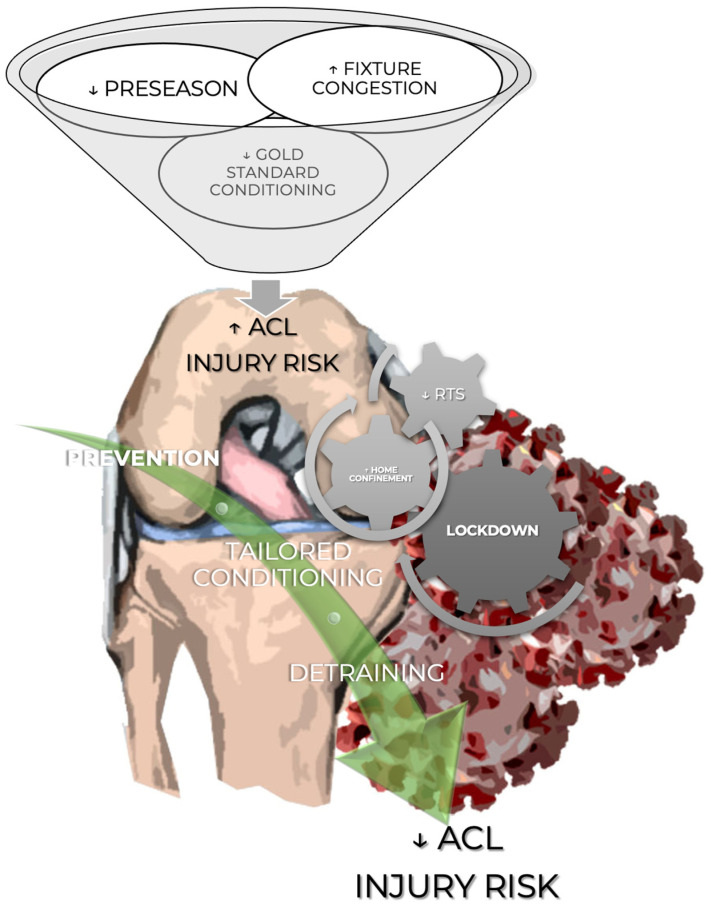
Assessment of the risk of anterior cruciate ligament injury during the COVID-19 pandemic.

Taken together, specific prevention programs that incorporate multimodal approaches have been shown to decrease the risk of ACL injuries (see Figure 1).

To successfully decrease the incidence rate of ACL ruptures, more emphasis must be placed on implementing these intervention strategies as a standard requirement in sports at every level of competition.

## 6. Impact of Rehabilitation and Telerehabilitation after ACL Reconstruction during the COVID-19 Pandemic

The COVID-19 pandemic has changed the rehabilitation needs of subjects, as shown by the REH-COVER action of the Cochrane Rehabilitation [58,59,60]. In this context, the rehabilitation process of patients undergoing ACL reconstruction surgery was a challenge for all healthcare professionals influenced by this pandemic [61,62]. In fact, short-term delayed surgeries are procedures that can wait for weeks, but there are literature studies that support increased pathology when waiting more than 3 months, such as with ACL reconstruction, myelopathy, or nerve compression with worsening symptoms or muscle weakness [63]. The impossibility of carrying out treatments during the closure of numerous rehabilitation wards or their transformation into COVID-19 wards or the contagion from COVID-19 were the most frequent problems to overcome [64]. The implementation of telemedicine services has represented an opportunity to overcome barriers to in-presence rehabilitation. In this context, telerehabilitation is characterized by rehabilitation services delivery through the support of new communication technologies [65,66].

Interestingly, telerehabilitation can be provided through programs, videos, or dedicated websites in synchronous mode (in real-time), asynchronous with supporting materials such as videos, or live in association with programming of activities carried out in asynchronous. During the COVID-19 pandemic, this innovative methodology represented an opportunity to enhance rehabilitation services [65,66].

In this context, a recent study by Lee et al. [67] assessed the postoperative care of patients undergoing arthroscopic anterior cruciate ligament reconstruction during the early phase of the pandemic. The authors reported that telehealth follow-up may be appropriate for ACL reconstruction patients beyond the pandemic without short-term differences in outcome measures compared to the standard approach. Similar results were shown by Bauwens et al. [68], who recently compared a self-rehabilitation smartphone app to standard in-person rehabilitation; the authors highlighted similar results between groups after 6 months, with significant improvements in telerehabilitation group [69].

To assess patients’ self-perceived effects, Dunphy and Gardner [69] surveyed one hundred patients who had undergone ACL surgery and a comprehensive rehabilitation treatment including telerehabilitation. Interestingly, the authors reported a high level of acceptability of telerehabilitation, suggesting potential implications for future interventions integrating technological advances in ACL injury rehabilitation.

In line with these findings, a study by Hong et al. [70] reported intriguing implications for integrating telemonitoring devices in home-based rehabilitation programs. Interestingly, the authors included 15 patients undergoing ACL reconstruction followed by a home-based rehabilitation program telemonitored by a knee brace with a motion tracker, a mobile app, and a web portal. Patients completed the rehabilitation exercise with the support of audio guidance and the real-time tracking system, which displayed the achieved motions on the user interface of the app. Six months after surgery, the authors reported that the home-based rehabilitative knee brace system might be considered a viable option to strictly assess knee muscle strengths and achieve knee range of motion outcomes [70]. Moreover, Fayard et al. [71] reported that self-rehabilitation application focusing on flexion contracture control and quadriceps recovery in the first 6 weeks after ACL reconstruction provides similar results to a rehabilitation protocol by an independent physiotherapist.

Despite these considerations, there is still a large knowledge gap about the long-term effects of integrating digital solutions in the comprehensive rehabilitation management of ACL injuries. Moreover, to our knowledge, no previous study assessed the effects of telerehabilitation programs prior to the surgical approach. However, it should be noted that growing evidence supports the role of rehabilitation in improving surgical outcomes when administered before surgical intervention [72]. In addition, the current literature suggests that telerehabilitation and telemonitoring devices could be effectively integrated into routine clinical practice of patients with ACL injuries, frequently characterized by young ages and high compliance rates to technological advances [73]. These results might be particularly intriguing considering the advantages of telerehabilitation, which might be considered a sustainable therapeutic strategy implementing standard rehabilitation and overcoming barriers related to reduced financial resources [74]. Lastly, Weaver et al. [6] investigated an age-related influence, but that study concluded that no group differences were observed for other isometric strength outcomes, Pedi-IKDC, or ACL-RSI scores for adolescents who underwent surgery pre-COVID versus during the COVID-19 pandemic timeframe, at three months after ACLR.

However, to date, there is still a large knowledge gap about the sanitary costs of specific telerehabilitation interventions, and studies assessing the cost-effectiveness of telerehabilitation in patients with ACL injury are still lacking.

Taken together, these findings suggested that technological advances and digital solutions might be considered promising tools to implement a comprehensive rehabilitation approach in patients with ACL injuries. Future research should focus on the long-term consequences of the implementation of digital solutions in ACL injury rehabilitation, underlining the potential advantages of integrating cutting-edge technology in the sustainable management of athletes with ACL injuries.

## 7. Conclusions

The COVID-19 pandemic has crucially affected the standard care of patients with ACL injury, with detrimental consequences on surgical intervention rate and rehabilitation treatment. Although in 2020, the amount of ALCR surgery was lower than predicted, the rate is currently increasing worldwide. Although the reasons are far from fully characterized, COVID-19-related fatigue, neuropathy, and myopathy might play a role. Despite the pandemic issues, it has nevertheless highlighted the role of telerehabilitation, which today can be considered an approach to be integrated into the conventional treatment process to facilitate follow-ups and proximity between healthcare professionals and patients.

Taken together, rehabilitation approaches might be effective in reducing ACL injury risk, targeting muscle strength unbalance, and altering neuromuscular control through multimodal programs. Moreover, the results of the present scoping review showed promising effects of telerehabilitation in patients after ACL reconstruction, with potential implications for integrating digital innovation in the tailored management of patients with ACL injury.

However, future studies are needed to provide additional evidence supporting a comprehensive approach integrating home-based and standard rehabilitation after ACLR.

## Data Availability

Not applicable.
